# Establishment and evaluation of a specific antibiotic-induced inflammatory bowel disease model in rats

**DOI:** 10.1371/journal.pone.0264194

**Published:** 2022-02-22

**Authors:** Guojun Tong, Hai Qian, Dongli Li, Jing Li, Jing Chen, Xiongfeng Li

**Affiliations:** 1 Departments of General Surgery, Huzhou Central Hospital, Huzhou, Zhejiang, China; 2 Central Laboratory, Huzhou Central Hospital, Huzhou, Zhejiang, China; 3 Orthopedic Surgery, Huzhou Central Hospital, Huzhou, Zhejiang, China; University of Illinois at Chicago, UNITED STATES

## Abstract

Physical and chemical methods for generating rat models of enteritis have been established; however, antibiotic induction has rarely been used for this purpose. The present study aimed to establish and evaluate a rat model of inflammatory bowel disease (IBD) using antibiotics. A total of 84 Sprague-Dawley (SD) rats were divided into the following groups, according to the dosage and method of administration of the antibiotics: A, control; B, low-dose clindamycin; C, medium-dose clindamycin; D, high-dose clindamycin; E, low-dose clindamycin, ampicillin and streptomycin; F, medium-dose clindamycin, ampicillin and streptomycin; and G, high-dose clindamycin, ampicillin and streptomycin. Antibiotic administration was stopped on day 7; the modeling period covered days 1–7, and the recovery period covered days 8–15. Half of the animals were dissected on day 11, with the remaining animals dissected on day 15. Food and water intake, body weight and fecal weight were recorded. Intestinal flora was analyzed via microbial culture and quantitative PCR. The content of TNF-α, IL1-β, IL-6 and C-reactive protein (CRP) was assessed in abdominal aorta blood. Colonic and rectal tissues were examined pathologically via hematoxylin-eosin staining to assess leukocyte infiltration and intestinal mucosal changes as indicators of inflammation. Rat weight, food intake, water intake and 2-h fecal weight were significantly different across the experimental groups (P = 0.040, P = 0.016, P<0.001 and P = 0.009, respectively). Microbial cultures revealed no significant differences between group A and B,C (P = 0.546,0.872) but significant differences betwenn group A and the other experimental groups (all P<0.001). Furthermore, significant differences in the levels of *Bacteroides*, *Faecalibacterium prausnitzii* and *Dialister invisus* on day 4 between groups A, C and F (P = 0.033, P = 0.025 and P = 0.034, respectively). Significant differences were detected in the levels of TNF-α, IL1-β, IL-6 and CRP between the groups (all P<0.001). The colonic and rectal pathological inflammation scores of the experimental groups were significantly different compared with group A (B vs. A, P = 0.002; others, all P<0.001). These findings indicated that an antibiotic-induced IBD model was successfully established in SD rats; this animal model may serve as a useful model for clinical IBD research.

## Introduction

Inflammatory bowel disease (IBD) includes Crohn’s disease and ulcerative colitis (UC), which are common intestinal diseases that are increasing in incidence each year [1]. Clinically, patients with IBD exhibit a slow treatment effect and a long treatment period [2]. IBD is considered a disorder of the immune system [3]. Changes in the intestinal flora and its metabolites affect the patient’s immune system, thereby inducing disease [4, 5]. IBD frequently involves activation of CD4+ T cells and dysregulation of TNF-α levels in circulating blood [6]; additionally, the disease commonly involves increased production of proinflammatory cytokines, such as IL-1β and IL-6 [7]. Furthermore, C-reactive protein (CRP) has been observed to be upregulated in patients with IBD, rendering it a candidate biomarker for detecting IBD [8]. In order to obtain in-depth insight into the disease, animal models, such as rats, have been utilized [9–12]. Sprague-Dawley (SD) rats can be raised and controlled easily due to their mild temperament; hence, they have been used extensively for the establishment of various disease models [13]. Rat IBD models are primarily induced using chemical factors such as 2,4,6-trinitrobenzene sulfonic acid (TNBS) and dextran sodium sulfate (DSS) [11, 12, 14, 15]. These models are easy to induce with high reproducibility, and can simulate the acute and chronic inflammatory processes that occur in UC; however, they do not fully simulate UC lesions, and there are challenges associated with dosing when inducing these models [16]. Physical methods such as chemical enema or physical radiotherapy typically induce inflammation of the animal’s rectum or sigmoid colon instead of total colorectal inflammation, and may cause mucosal and muscle acute necrosis; therefore, they do not accurately simulate UC or IBD observed in the clinic, limiting their utility [17]. Therefore, it is necessary to explore new methods of IBD modeling.

As imbalances in the intestinal flora may lead to enteritis, this has been used as a basis for modeling [18, 19]. Antibiotics frequently induce imbalances in the intestinal flora [20, 21]. Intestinal microbes are sensitive to clindamycin, ampicillin and streptomycin; thus, these antibiotics can easily cause intestinal microbial imbalance [22–27]. Therefore, the present study used different doses of clindamycin, as well as different combined doses of clindamycin, ampicillin and streptomycin, to disrupt the intestinal flora in rats. Inflammatory factors in the abdominal aorta, and the inflammation of the colon and rectum were analyzed to evaluate the utility of the model.

## Materials and methods

### Ethics

This study follows the Basel Declaration of 2010 and Institutional Animal Care and Use Committee (IACUC) of Xi’an United Nations Quality Detection Technology Co., Ltd. Laboratory (no reference number). All applicable international, national, and/or institutional guidelines for the care and use of animals were followed.

### Rats

SD female rats (n = 84; age, 5–6 weeks; weight, 172.4–179.5 g) were obtained from Liaoning Changsheng Biotechnology Co., Ltd. All animals were quarantined and fed using a specific pathogen-free barrier system in the Xi’an United Nations Quality Detection Technology Co., Ltd. Laboratory for 9 days. All procedures were performed in accordance with the National Institute of Health Guide for the Care and Use of Laboratory Animals [28]. All experimental procedures and animal handling were performed in accordance with the guidelines of the International Association for the Study of Pain, and the animal protocols were approved by the Xi’an United Nations Quality Detection Technology Co., Ltd. Animal Committee. The authors made an effort to minimize the number of animals used.

### Experimental drugs, reagents and instruments

The drugs used in the present study were as follows: Clindamycin hydrochloride (cat. no. C10038638; Shanghai Macklin Biochemical Co., Ltd.), 99% purity; ampicillin (cat. no. L220S26; Hebei Bailingwei Superfine Material Co., Ltd.), 98% purity; and streptomycin (cat. no. 20171123; Tianjin Guangfu Fine Chemical Research Institute), ≥90% purity.

Rat IL-1β (rat.no.20884), IL-6 (YPH102698), TNF-α (EK0526) and C-reactive protein (CRP) ((cat. no. 201810).ELISA kits were obtained from Bioswamp Life Science LabA soil genomic DNA rapid extraction kit, rapid competent cell preparation kit (one-step method), SanPrep column plasmid DNA small amount extraction kit (cat. no. B518191-0050) and SanPrep column DNA gel recovery kit (cat. no. B518131-0050) were all purchased from Sangon Biotech Co., Ltd. Taq Plus DNA polymerase (cat. no. B600090), agarose B (cat. no. A600014) and 4S Red Plus Nucleic Acid Stain (10,000X aqueous solution; cat. no. A606695) were from BBI (Sangon Biotech Co., Ltd.). GeneRuler DNA Ladder Mix was procured from Thermo Fisher Scientific, Inc. A pMD™18-T Vector Cloning kit was obtained from Takara Biotechnology, Co., Ltd.

Bacteroides-Bile-Enterprise (BBE) agar (cat. no. 20171215; Qingdao Hope Bio-Technology Co., Ltd.), *Lactobacillus* selective agar (cat. no. 20180424; Qingdao Hope Bio-Technology Co., Ltd.), anaerobic bacteria agar (cat. no. 171209; Beijing Luqiao Technology Co., Ltd.), trypticase-phytone-yeast (TPY) agar medium (cat. no. 180208; Beijing Luqiao Technology Co., Ltd.), mannitol sodium chloride agar medium (cat. no. 171008; Beijing Luqiao Technology Co., Ltd.), eosin methylene blue (EMB) agar (cat. no. 160504; Beijing Luqiao Technology Co., Ltd.), CATC agar (cat. no. 20170902; Qingdao Hope Bio-Technology Co., Ltd.), and reinforced *Clostridium* culture medium (cat. no. 171117; Beijing Luqiao Technology Co., Ltd.) were purchased for bacterial culture.

An Electric Day constant temperature incubator was purchased from Tianjin Taisite Instrument Co., Ltd. (cat. no. DH6000BII). A LabSystems Multiskan MS 352 microplate reader was purchased from Thermo Fisher Scientific, Inc. A low-speed condensation centrifuge was purchased from Shanghai Lu Xiangyi Centrifuge Instrument Co., Ltd (cat. no. DDL-5M). An upright microscope was purchased from Nikon Corporation.

The following instruments were utilized for quantitative (q)PCR: High-speed refrigerated centrifuge (cat. no. HC-2518R; Anhui Zhongke Zhongjia Science Instrument Co., Ltd.); electrophoresis instrument (cat. no. DYY-6C; Beijing Liuyi Biotechnology Co., Ltd.); electrophoresis tank (cat. no. H6-1; Shanghai Jingyi Plexiglass Products Instrument Factory); gel imaging system (cat. no. FR980; Shanghai Furi Science & Technology Co., Ltd.); micro spectrophotometer (cat. no. SMA4000; Merinton Instrument, Inc.); PCR machine (Bio-Rad Laboratories, Inc.); and a generation sequencer and StepOne™ fluorescence quantitative PCR instrument (Applied Biosystems; Thermo Fisher Scientific, Inc.).

### Animal grouping and modeling

**Fig 1:**
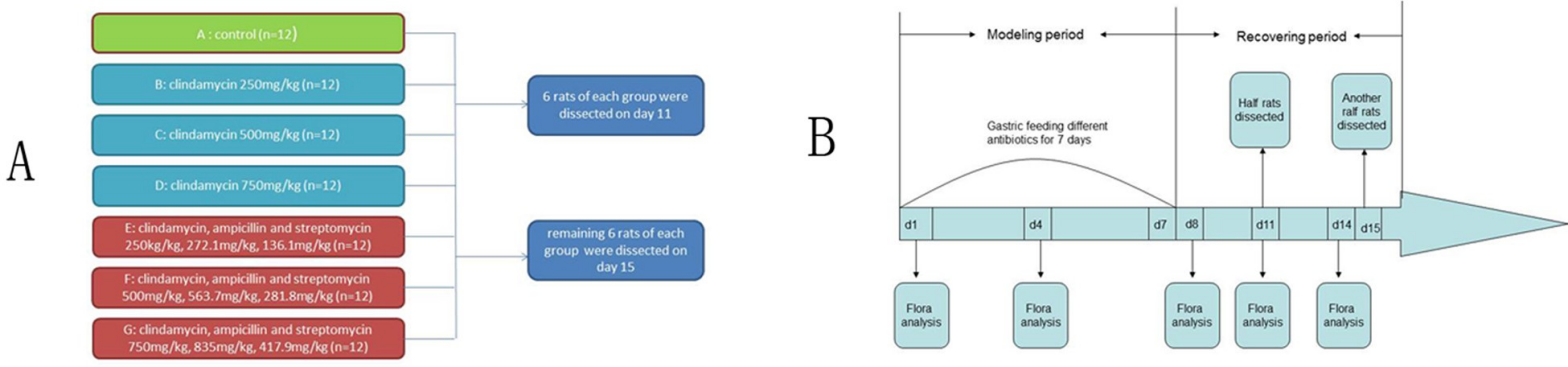
Study flow and method. (A) Group design of the study. (B) Experimental work flow of the study.

The animals were grouped as follows: A, control group, normal saline; B, low-dose clindamycin group (250 mg/kg); C, medium-dose clindamycin group (500 mg/kg); D, high-dose clindamycin group (750 mg/kg); E, low-dose triple antibiotic group (clindamycin, ampicillin and streptomycin; 250, 272.1 and 136.1 mg/kg, respectively); F, medium-dose triple antibiotic group (clindamycin, ampicillin and streptomycin; 500, 563.7 and 281.8 mg/kg, respectively); and G, high-dose triple antibiotic group (clindamycin, ampicillin and streptomycin; 750, 835.8 and 417.9 mg/kg, respectively). The experiment was divided into two stages: The modeling period (days 1–7) and the recovery period (days 8–15). The administration volume was 10 ml/kg/day intragastrically using an oral needle during the modeling period between 8:30–10:00 a.m. The intragastric administration was stopped on day 8. Animal weight, and food and water intake were monitored, and stool samples were collected on days 1, 3, 5, 7, 9, 11 and 14 within 2 h post-administration of antibiotics. For each rat, the fecal microbial flora was examined on days 1, 4, 8, 11 and 14. On days 11 and 15, under sodium pentobarbital anesthesia (intraperitoneal; 2%; 40 mg/kg), the abdominal walls of 50% of the animals in each group were cut, the abdominal aorta exposed and abdominal aortic blood drawn (Fig 1A and 1B). Then, the rats were dissected after being euthanized via exsanguination whilst under anesthesia by cutting the abdominal aorta, and death was confirmed by the pulsation of the abdominal aorta disappearing and the pupils dilating, after which colonic and rectal tissues were collected.

### Microbial culture

The microbial species detected were *Staphylococcus aureus*, *Bifidobacterium*, yeast, *Bacteroides*, *Clostridium*, anaerobic bacteria, *Escherichia coli* (*E*. *coli*), *Enterococcus and Lactobacillus*. An equivalent of 1 g feces was mixed with 9 ml tryptone soy broth. A volume of 20 μl of the appropriately diluted sample was spread evenly on mannitol sodium chloride, EMB and CATC agar plates and cultured under aerobic conditions at 37˚C for 48 h. The above organisms were also cultured on TPY, BBE, reinforced *Clostridium* medium, anaerobic and *Lactobacillus* selective agar plates at 37˚C for 48 h in anaerobic conditions. Organisms inoculated on dichloran rose-Bengal chloramphenicol agar plates were cultured for 5 days at 28˚C in aerobic conditions. The colonies were stained by methylene blue and enumerated using the following formula: Number of colonies (CFU/g) = number of plate colonies x 50 x dilution factor, with x10^6^ used as the uniform independent unit.

### Diarrhea within 2 h after administration

Within 2 h after drug administration on days 1, 4, 8, 11 and 14, the number of defecations of each rat in each group was counted to determine whether they exhibited diarrhea, as well as the severity of diarrhea if present. If >2 defecations were observed, then diarrhea was considered to be present. Additionally, changes in the diet and water consumption of rats were monitored. It was observed that the rats exhibited no obvious signs of dehydration and could tolerate the experimental protocol.

### qPCR analysis

A soil genomic DNA rapid extraction kit was used to extract fecal DNA from SD rats as follows: i) SD rat feces (400 mg) was mixed with 400 μl of 65˚C pre-warmed Buffer SCL via agitation in a 65˚C water bath for 5 min; ii) supernatant (350 μl) was obtained via centrifugation at 2,250 x g for 3 min at room temperature; iii) an equal volume of Buffer SP was mixed via inversion, and placed on an ice bath for 10 min; iv) centrifugation was conducted at 2,250 x g for 3 min at room temperature; v) the resulting supernatant was mixed with chloroform, followed by centrifugation at 2,250 x g for 3 min at room temperature; vi) the upper aqueous phase was mixed with 2 volumes of absolute ethanol via inversion and incubated at room temperature for 3 min, followed by centrifugation at 1570g(rcf) for 5 min at room temperature; vii) ethanol (75%; 1 ml) was added to the pellet for washing via centrifugation at 2,250 x g for 3 min at room temperature, with this step repeated; viii) the residual ethanol is allowed to evaporate; and ix) the resulting DNA pellet is solubilized in 70 μl TE Buffer. qPCR was performed using a pMD18-T Vector Cloning kit, Taq polymerase and a StepOne fluorescence quantitative PCR instrument. Of the aforementioned nine microbiota, only *Bacteroides* was successfully detected via PCR. Therefore, fecal matter was collected on day 4, and the presence of *Bacteroides*, *Faecalibacterium prausnitzii (F*. *prausnitzii)* and *Dialister invisus (D*. *invisus)* was analyzed via qPCR. Gene fragments from *Bacteroides*, *F*. *prausnitzii* and *D*. *invisus* were retrieved from the NCBI GenBank database (https://www.ncbi.nlm.nih.gov/genbank/); the primers used are presented in S1 Table. The amplicons were analyzed via 1.5% agarose gel electrophoresis and ethidium bromide was excited by standard 302nm ultraviolet transmittance. Bands were imaged using a DNA gel electrophoresis imager (Analytik Jena AG) and quantified using Quantity One v4.6.6 (Bio-Rad Laboratories, Inc.).

### Analysis of inflammatory factors

On days 11 and 15, half of the rats in each group were dissected under intraperitoneal anesthesia as aforementioned. The abdomen was cut, and 5 ml blood was withdrawn from the abdominal aorta. Blood serum (0.5 ml) was obtained from each sample via centrifugation at 1,570 x g for 3 min at 4˚C. The content of TNF-α (EK0526), IL-1β (rat.no.20884), IL-6 (YPH102698), and CRP (cat.no.2018) was detected in the blood serum without diluting via ELISA according to the manufacturer’s protocols.

### Colonic and rectal pathological inflammation assessment

We choose the relative same part of colon and rectum for pathological analysis on days 11 and 15. All tissues were stained using hematoxylin-eosin before inflammation assessment. The hematoxylin-eosin staining process were as follows: Tissues were fixed in 10% formaldehyde at room temperature for 10 min, embedded in paraffin and cut into sections (5–8 um). The sections were stained in hematoxylin for 5 min and alcohol eosin staining solution for 2–3 min at room temperature. An upright light microscope was used to analyze the stained tissues (magnification, x200).The degree of neutrophil infiltration and intestinal mucosal changes were graded as follows: 0, no inflammation; 1, low multifocal neutrophil infiltration [<10 neutrophils/high-powered field (HFP)]; 2, moderate multifocal neutrophil infiltration (submucosal involvement; 10-50/HPF); 3, extensive multifocal and aggregated neutrophil infiltration (submucosal involvement and muscle layer; >50/HPF); 4, additional abscesses or extensive muscle layer involvement [15, 29]. Each section was analyzed in three fields, and the score in each section was added to produce the final score.

### Statistical analysis

Primer Premier 5.0 software (Premier Biosoft International) was used for designing the primers. The data were analyzed using SPSS 21 software (IBM, Corp.). Continuous data were analyzed using one-way ANOVA followed by Tukey post hoc test, and are presented as the mean±SD. Ordinal data were analyzed using Kruskal-Wallis H test and Dunn’s post hoc test, and are presented as the median and interquartile range. P<0.05 was considered to indicate a statistically significant difference.

## Results and discussion

### Comparison of basic indices

**Fig 2:**
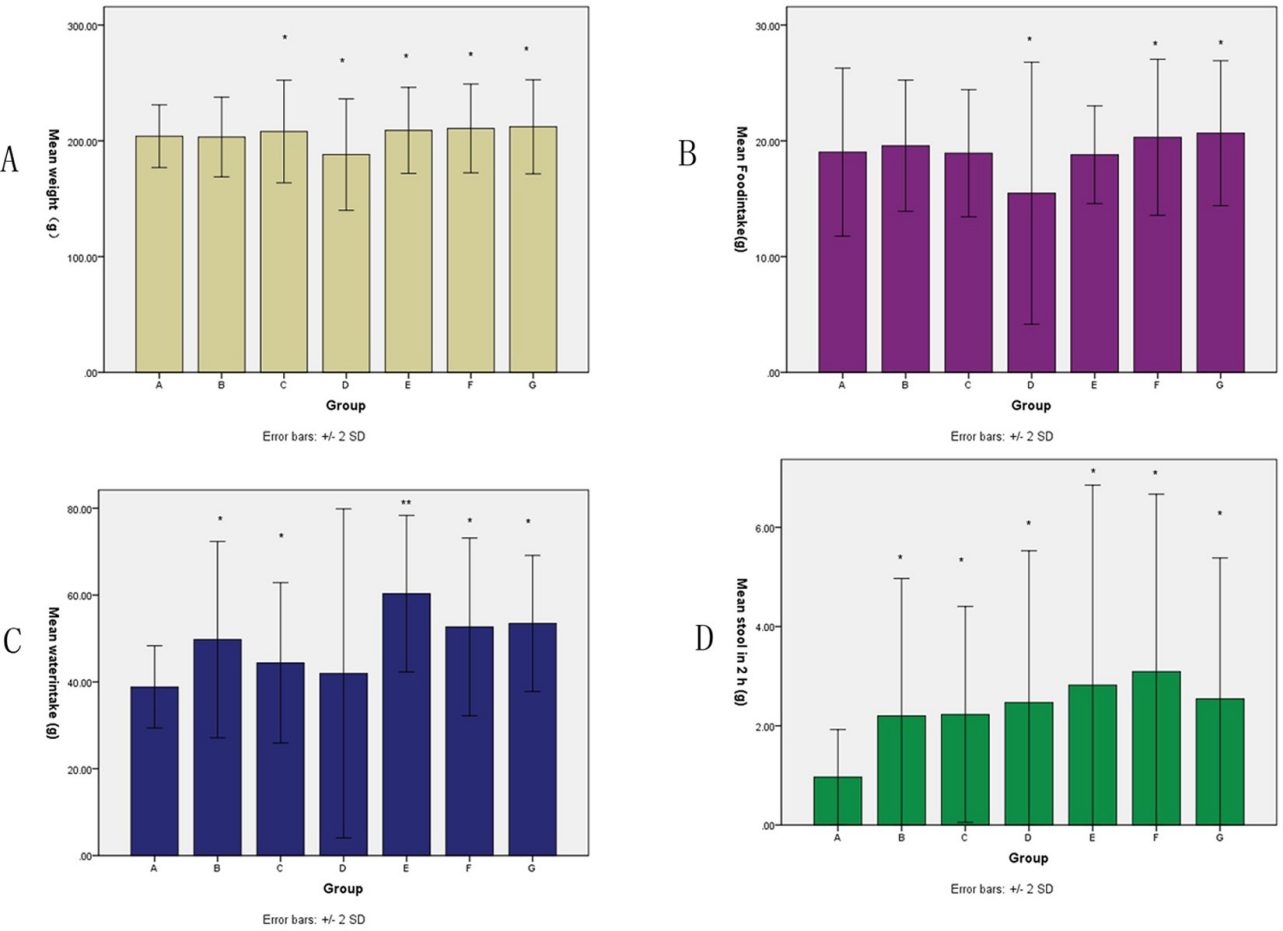
Mean basic characteristics on days 1,3,5,7,9,11,13,14. (A) Comparison of animal weights (B)Comparison of food intake of the animals (C) Comparison of water intake of animals (D) Comparison of stool within 2 h. Data are presented as the mean ± SD.Treatment groups vs control groups: * <0.05;**<0.001.

The present study aimed to establish an antibiotic-induced rat IBD model. Previous studies have conducted comparative research on rat IBD models induced using different chemical factors, and speculated the various advantages and disadvantages of models induced using agents such as TNBS and DSS [11, 12, 14]. IBD is a group of chronic inflammatory disorders that affect individuals throughout life. Although factors involved in the etiology and pathogenesis of IBD remain unknown, studies using animal models of colitis have indicated that dysregulation of host/microbial interactions is a requisite for the development of IBD [30–32].As we know, IBD usually causes changes in diet, weight and stool.The average starting weight of all rats was 172.6±2.49 g, and no significant difference was detected in the weight between groups A-G before experiment (P>0.05;). Significant differences between groups were observed for the mean weight, food intake, water intake and stool in 2 h post-administration on days 1,3,5,7,9,11,13,14 between groups and some significant difference were observed within groups (S2 Table and Fig 2). The mean weight, food intake, water intake and stool in 2h increases significantly not dependent on dosage and method of administration. These results may come from comprehensive calculation of different days.

### Comparison of nine microbes between groups

**Fig 3:**
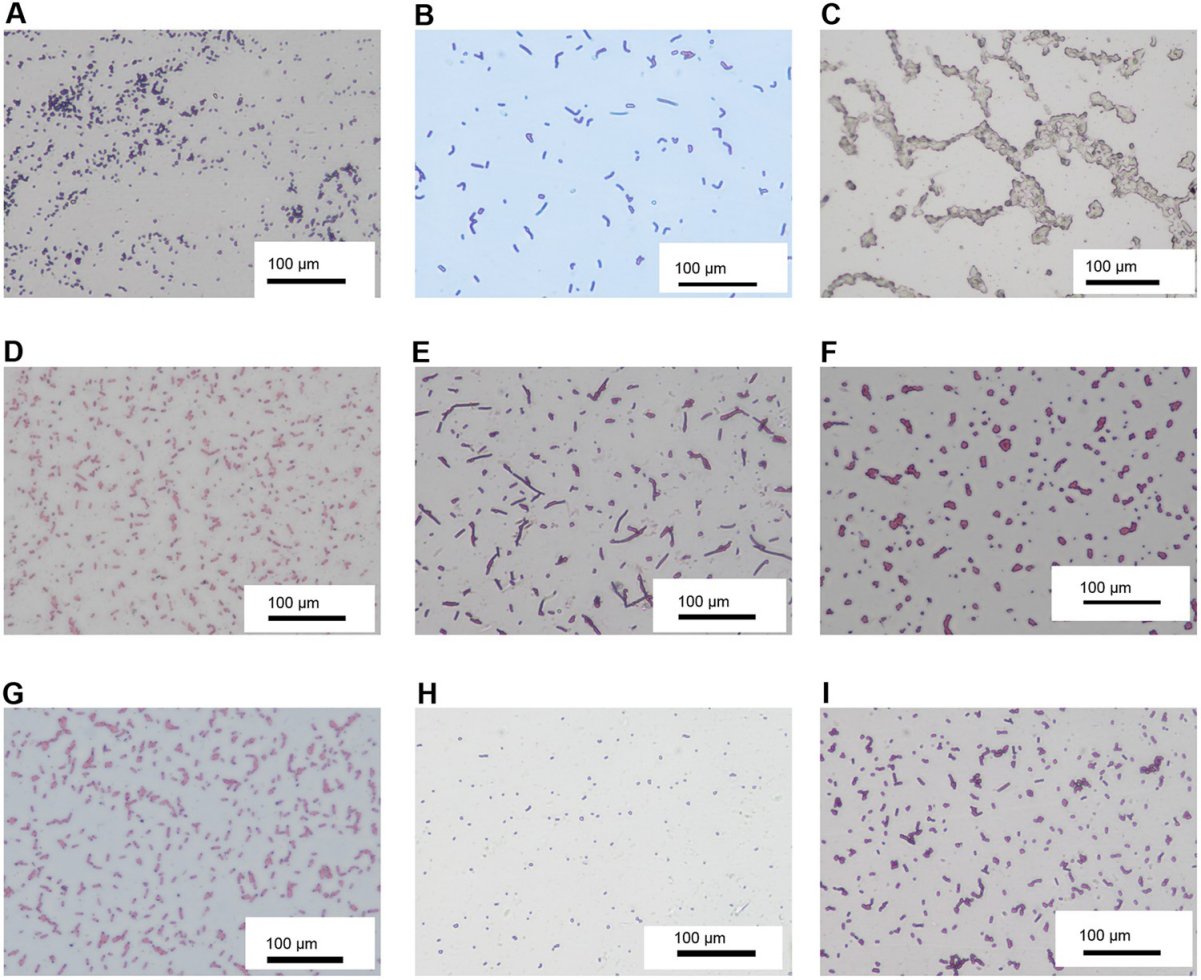
Microbial specimens from rat feces under a microscope on day 4 (methylene blue staining, x100). (A) *Staphylococcus aureus*, (B) *Bifidobacterium*, (C) yeast, (D) *Bacteroides*, (E) *Clostridium*, (F) anaerobic bacteria, (G) *Escherichia coli*, (H) *Enterococcus* and (I) *Lactobacillus*.

**Fig 4:**
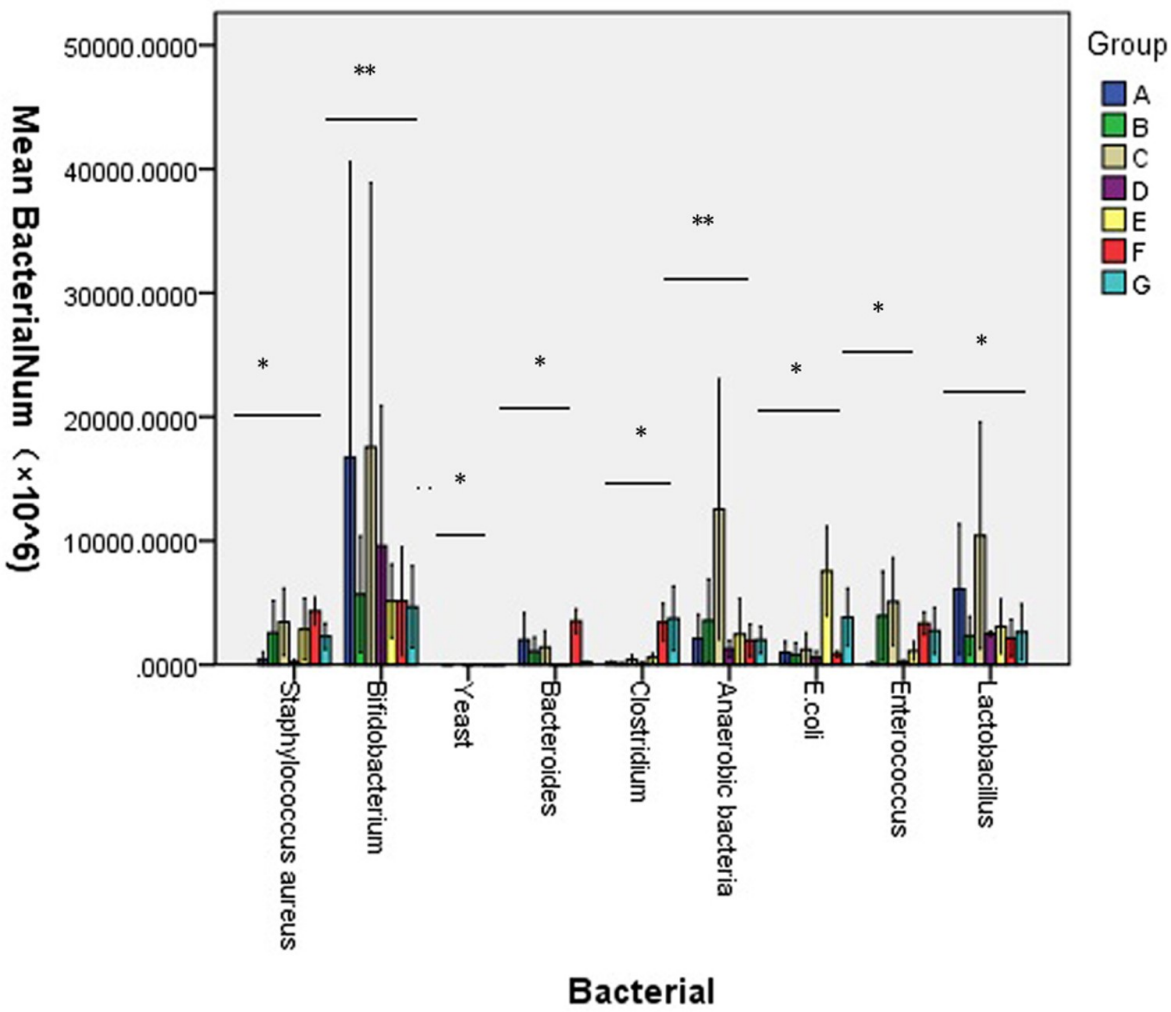
Comparison of nine types of microflora in the feces between A to G groups. The results show the levels of beneficial bacteria (*Bifidobacterium*, *Clostridium*, *Lactobacillus)* decreased significantly, while pathogenic bacteria (*S*. *aureus*, yeast, *Bacteroides*, anaerobic bacteria, *E*. *coli*, *Enterococcus*) levels increased significantly.* P<0.05,** P<0.001.

**Fig 5:**
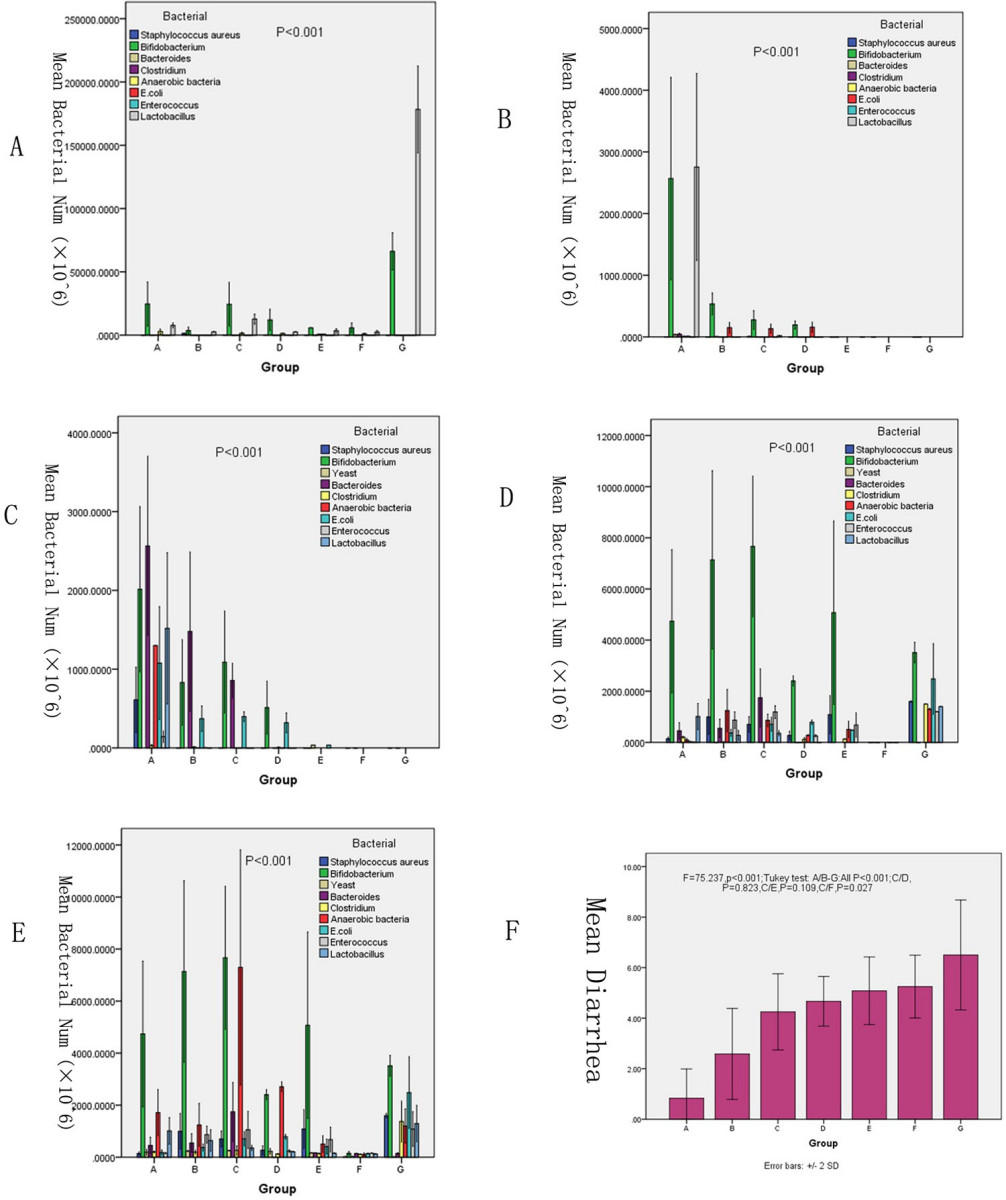
Nine types of microflora in the feces of experimental rats. (A)Comparison on day 1, (B) Comparison on day 4, (C)Comparison on day 8, (D) Comparison on day 11 and (E) Comparison on day 14. (F) Defecation of rats within 2 h of antibiotic administration across the study period. Data are presented as the mean ± SD.

*S*. *aureus*, *Bifidobacterium*, yeast, *Bacteroides*, *Clostridium*, *anaerobic bacteria*, *E*. *coli*, *Enterococcus and Lactobacillus* were cultured using special medium, and the number of colonies was counted [Fig 3]. Levels of beneficial bacteria (*Bifidobacterium*, *Clostridium*, *Lactobacillus)* decreased significantly, while pathogenic bacteria (*S*. *aureus*, yeast, *Bacteroides*, anaerobic bacteria, *E*. *coli*, *Enterococcus*) levels increased significantly (Fig 4). The total microbial load of the nine species was calculated and compared between the control and experimental groups (S3 Table). Microbial cultures revealed no significant differences between group A and B,C (P = 0.546,0.872) but significant differences between group A and the other experimental groups (all P<0.001) The mean levels of the nine microbes in each group were significantly different on days 1, 4, 8, 11 and 14 respectively (all P<0.001; Fig 5A–5E). A previous study utilized a similar method to study gut microbiota, finding that 4-week administration of an antibiotic cocktail depressed the ventilator response to hypercapnic stress in conscious animals. [33]. This study also found that microbiota-depleted rats exhibited decreased systolic blood pressure, and that chronic antibiotic intervention or fecal microbiota transfer resulted in disruptions to brainstem monoamine neurochemistry that were associated with the abundance of various bacteria. In the present study, changes in the 9 microbiota in different days were obseverved. *S*. *aureus* is a major human pathogen that causes a wide range of clinical infections [34, 35]. *Bifidobacterium* is a group of living microorganisms commonly included in supplements that confer health benefits on the host when administered in sufficient quantities [36]. *Bifidobacterium* levels were significantly reduced in the experimental groups compared with the control group.(Fig 4) *Yeast* is often employed in industrial fermentation processes due to its ability to efficiently convert relatively high concentrations of sugars into ethanol and carbon dioxide [37]. *Yeast* is not a common intestinal bacterium and was absent in the control group; it was only detected in the low- and medium-dose triple antibiotic groups. *Bacteroides* is a gram-negative, non-spore, obligate anaerobic bacillus [38, 39]. *Clostridium* species are anaerobic, gram-positive, rod-shaped, endospore-forming bacteria belonging to the phylum *Firmicutes*, and they constitute both a class and a genus within the phylum [40]. *Clostridium* levels decreased in the single-agent groups, but increased in the combined drug group.(Fig 4) Anaerobic bacteria serve pivotal roles in the microbiota of humans; these are also infectious agents involved in various pathological processes such as ChuW, ChuX and ChuY which were contiguous genes downstream from a single promoter that were expressed in the enteric pathogen.[41]. Pathogenic variants of *E*. *coli* cause substantial morbidity and mortality worldwide [42]. *Enterococcus* strains adhere strongly to the intestinal epithelium, forming biofilms, and possess antioxidant defense mechanisms that affect inflammatory processes [43]. The genus *Lactobacillus* consists of 173 species, evolutionary relationships between lactobacilli can be analyzed using core-genome trees [44]. Due to their symbiotic roles in humans, when lactobacilli mutate, they can produce infectious diseases such as colpitis and pelvic infection [45]. Antibiotic-induced gut dysbiosis induces various effects on the body. Liu *et al* [46] and Yu *et al* [47] considered the effects on body metabolism. Other studies reported associated effects on the nervous system [48–50]. Additional studies identified effects on tumor progression [51, 52]. Additional effects in other physiological systems, such as the immune system [53] and intestinal barrier [54, 55], have been reported. Due to these various possible effects, antibiotic-induced gut dysbiosis requires further investigation. In the present study, with increasing dose of antibiotics and enhanced effect when antibiotics were combined, levels of beneficial bacteria (*Bifidobacterium*, *Clostridium*, *Lactobacillus)* decreased significantly, while pathogenic bacteria (*S*. *aureus*, yeast, *Bacteroides*, anaerobic bacteria, *E*. *coli*, *Enterococcus*) levels increased significantly.

### Diarrhea analysis

The number of defecations within 2 h after drug administration were counted for each rat in each group across all included days (days 1, 4, 8, 11 and 14; S4 Table). Mean defecations in groups A-G across all included days were 0.833±0.578, 2.583±0.900, 4.250±0.754, 4.667±0.492, 5.083±0.669, 5.250±0.622 and 6.500±1.087, respectively. Using one-way ANOVA and Tukey post hoc test, it was observed that all experimental groups exhibited significantly greater defecation than group A (all P<0.001; Fig 5F). IBD often results in diarrhea [56]. The present study showed that all doses and antibiotic treatments induced significant diarrhea compared with the control group. Patients with IBD often sufferer from diarrhea; the present study observed that diarrhea was exacerbated with increased dose. It is proposed that the extent of diarrhea was related the degree of intestinal microbiota disorder and intestinal inflammation.

### qPCR analysis of three strains

**Fig 6:**
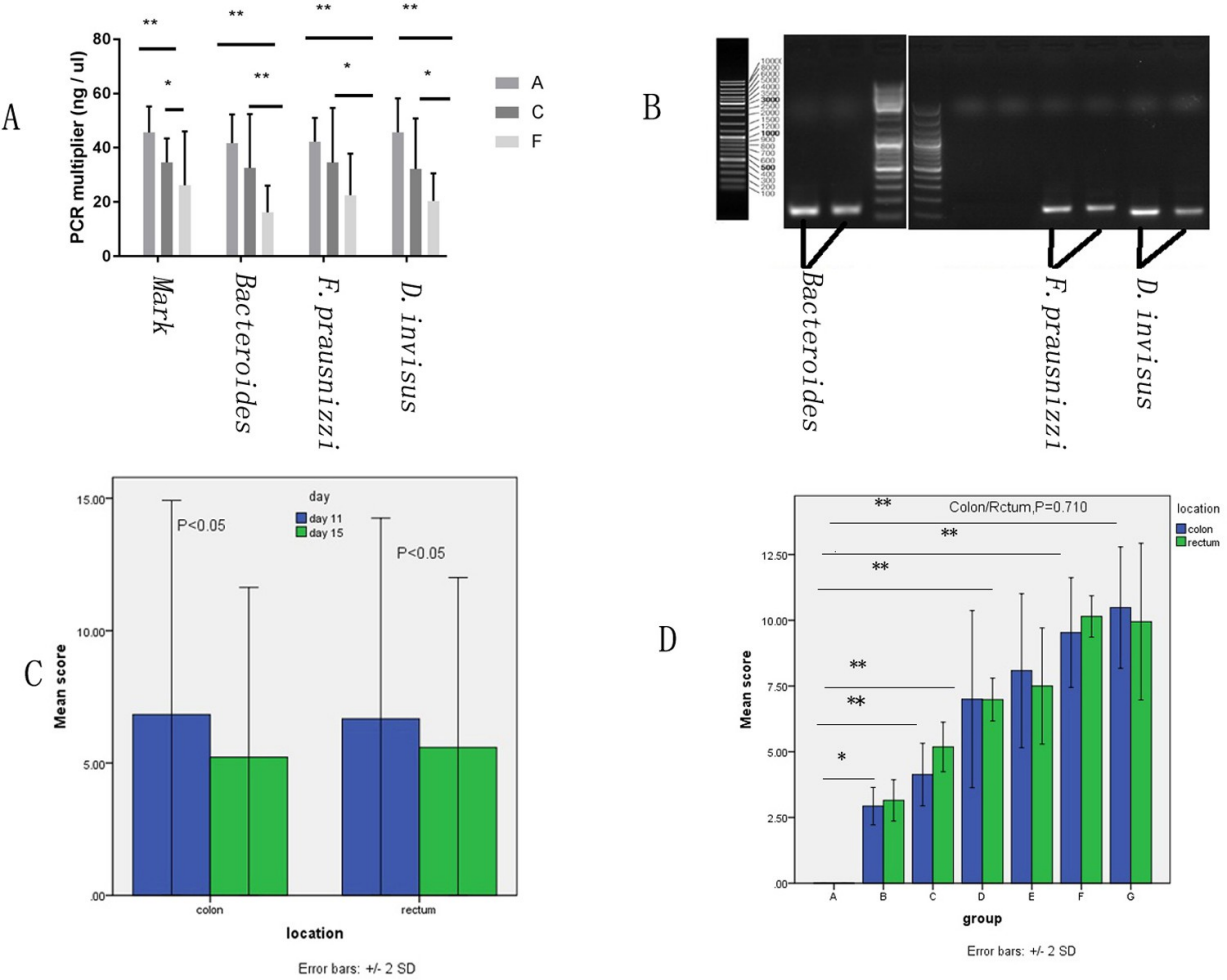
Fecal bacterial content and gut inflammation scores. (A) Comparison of bacterial amplification via quantitative PCR in groups A, C and F;(B) *Bacteroides*, *F*. *prausnitzii* and *D*. *invisus* agarose electrophoresis; (C)Comparison of colonic and rectal tissue inflammation scores on day 11 and day 15;(D)Comparison of colonic and rectal tissue inflammation scores of control group vs experiment groups respectively,*P<0.05, **P<0.01.

The concentrations of DNAmarks, *Bacteroides*, *F*. *prausnitzii* and *D*. *invisus* in A,C,F groups on fourth day were compared via qPCR. The same DNA marks expressed differently in the three gene fragments. One-way ANOVA revealed significant effects of treatment on the levels of DNA mark (P = 0.044), *Bacteroides* (P = 0.036), *F*. *prausnitzii* (P = 0.027) and *D*. *invisus* (P = 0.036). Post hoc analysis revealed significant decreases in quantity of *Bacteroides*, *F*. *prausnitzii* and *D*. *invisus* between group F and group A (all P<0.01), as well as between group F and group C (P<0.01 for *Bacteroides*, otherwise P<0.05; Fig 6A and 6B, S1 Raw Image). *Bacteroides*, *F*. *prausnitzii* and *D*. *invisus* are associated with IBD [57–59]. Thus, qPCR analysis of these three strains was performed in groups A, C and F, after attempts to perform qPCR analysis of the aforementioned nine types of microbiota were unsuccessful. The results showed that decreased quantities of these bacteria were observed following combination antibiotic treatment compared with clindamycin alone. *Bacteroides* species constitute a large part of the human gut microbiota, including both probiotics and pathogenic bacteria (depending on various genetic and environmental factors), and can cause disease conditions as varied as skin infections, endocarditis, intra-abdominal sepsis, appendicitis, bacteremia, pericarditis, meningitis and brain abscesses [60–62]. There is currently increasing interest in *F*. *prausnitzii*, one of the most abundant enteric microorganisms. However, whether there are other strains within this species and whether there are other *Faecalibacterium* species remain unclear [63, 64]. *D*. *invisus* levels are low or absent in patients with IBD [65]. The present study showed that *D*. *invisus* was only observed in small quantities in rat feces, with smaller levels in model groups.

### Changes in abdominal aortic inflammatory factors

**Fig 7:**
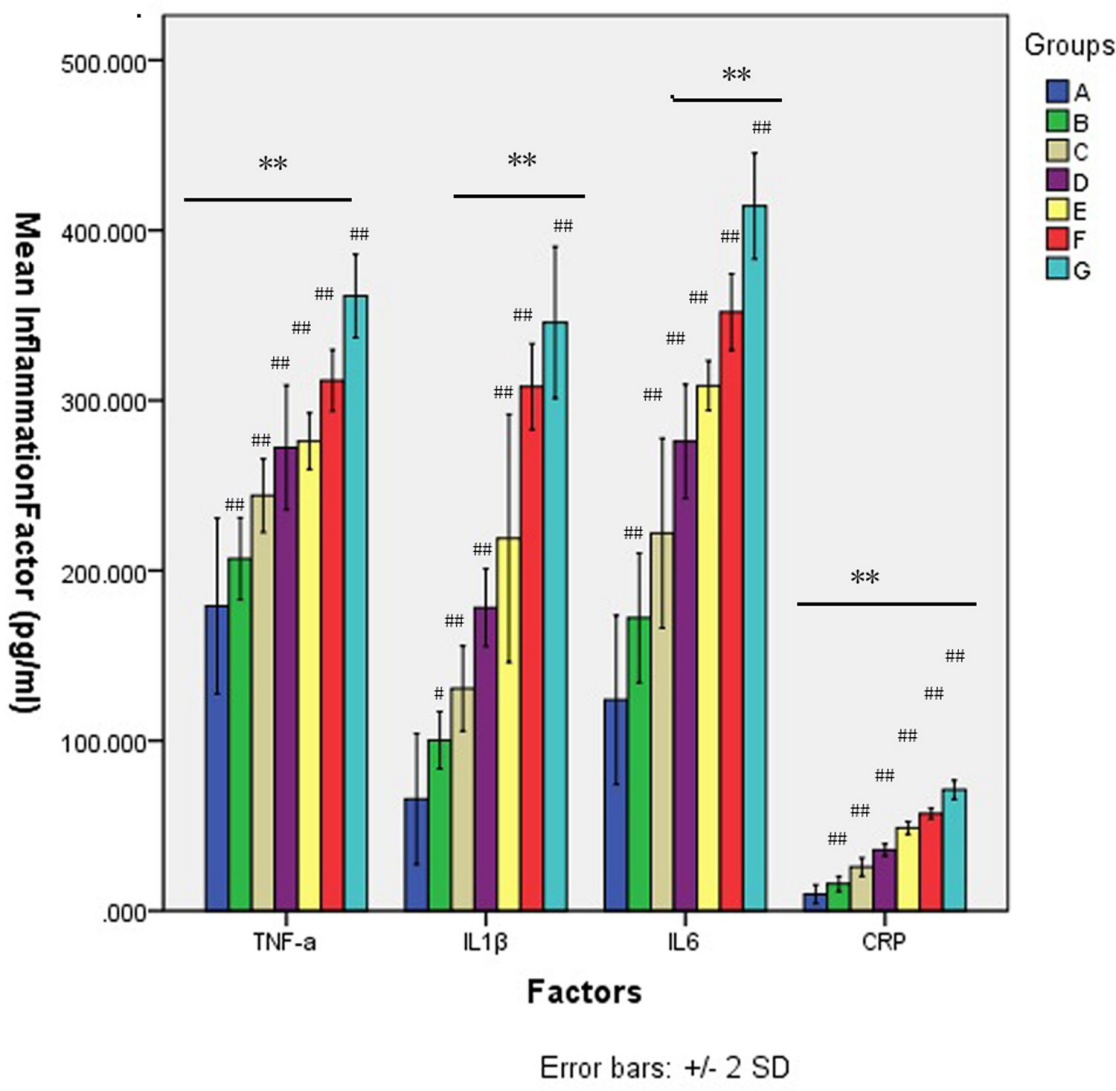
Comparison of inflammation factors between groups and within groups. **P<0.001, Comparison between groups; # = 0.001,##<0.001,Comparison for experiment groups vs control groups.

The levels of TNF-α, IL1-β, IL-6 and CRP in serum from the abdominal aorta were evaluated, and one-way ANOVA revealed significant effects of group type on the levels of these factors (All P<0.001; S4 Table, Fig 7). Post hoc analysis revealed significant increases in the levels of all these factors in the experimental groups compared with group A (P = 0.001, group B vs. group A for IL-1β; others, all P<0.001). The present study found that abdominal aortic blood levels of TNF-α, IL-1β and IL-6 were significantly upregulated in all groups compared with the control group. TNF-α has been reported to be a potent stimulator of IL-6 production [66–68]. Inflammation induces IL-1β production in Kupffer cells and hepatocytes [69]. CRP is increasingly studied within the context of inflammation and associated diseases [70–72]. The present study found that elevated CRP was detected in groups that received antibiotics compared with the control group.

### Comparison of pathological inflammation scores

**Fig 8:**
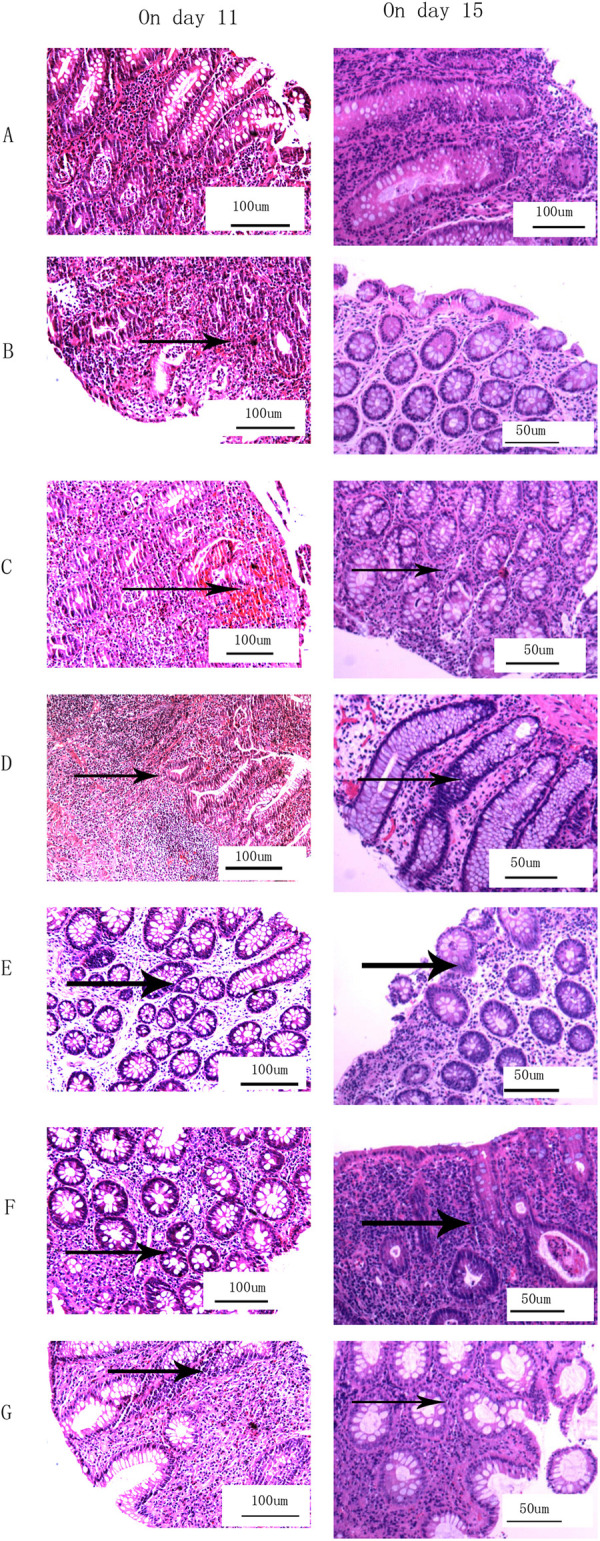
Pathological images of colonic hematoxylin-eosin staining. Images from groups (A) A, (B) B, (C) C, (D) D, (E) E, (F) F and (G) G. Left, rat colon tissue on day 11; right, rat colon tissue on day 15. In group A, no obvious neutrophil infiltration and mucosal edema were observed. In groups B-G, neutrophil infiltration was severe, and marked mucosal edema was notable; additionally, mucosal necrosis was observed, and focal ulcers were formed.

**Fig 9:**
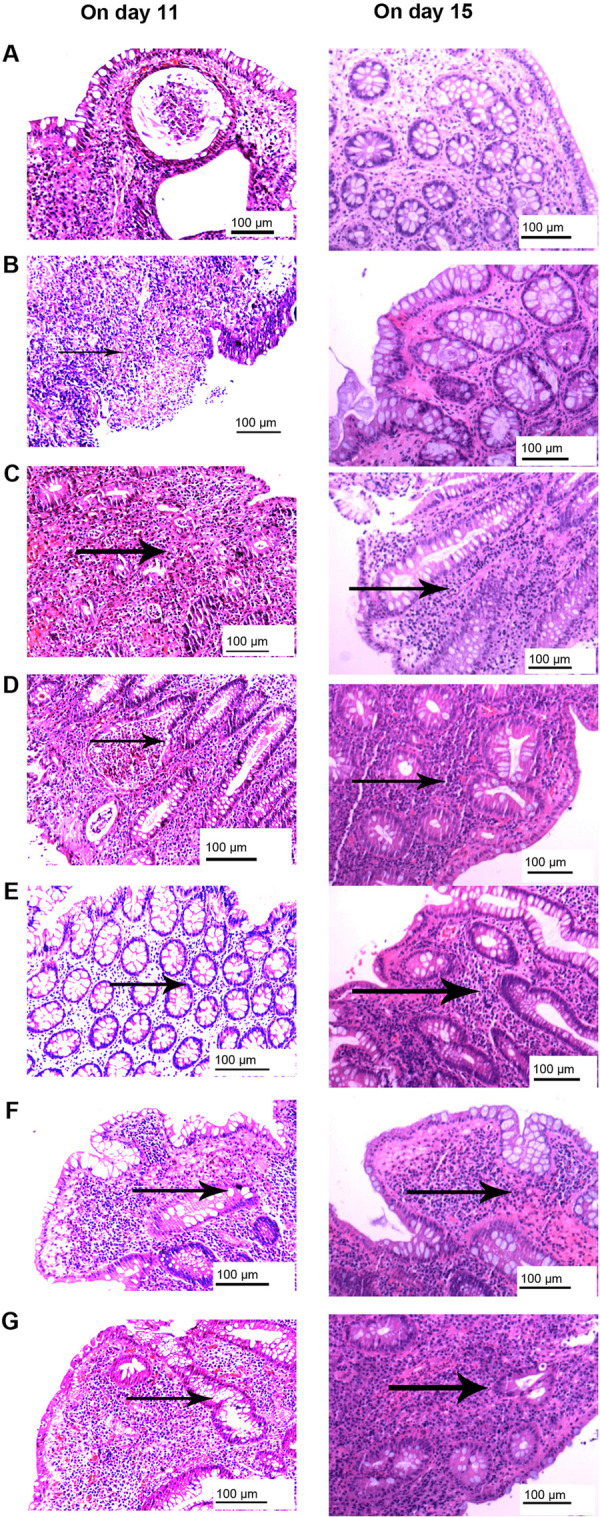
Pathological images of rectal tissue hematoxylin-eosin staining. Images from groups (A) A, (B) B, (C) C, (D) D, (E) E, (F) F and (G) G. Left, rat rectum tissue on day 11; right, rat rectum tissue on day 15. In group A, no obvious neutrophil infiltration and mucosal edema were observed. In groups B-G, neutrophil infiltration was severe, and marked mucosal edema was notable; additionally, mucosal necrosis was observed, and focal ulcers were formed.

Colonic and rectal tissues were scored for pathological inflammation after hematoxylin-eosin staining. These data were analyzed using Kruskal-Wallis H test followed by Dunn’s post hoc test. Significant differences in inflammation were observed between group A and all experimental groups (P = 0.002 for group B vs. group A, all other comparisons vs. group A, P<0.001; S5 Table). We found that pathologic inflammation sores increased with the increasing of dosage and combined administration.(Fig 6C and 6D) However, no significant difference was detected in the degree of inflammation between the two tissues (P = 0.710; Fig 6D). Additionally, inflammation scores were significantly higher on day 11 compared with day 15 within groups for both colonic and rectal tissues (Fig 6C). Pathologic colonic and rectal sections for control group and different experimental groups are presented in Figs 8 and 9. Pathological examination is important for evaluating the degree of tissue inflammation. Colonic and rectal tissues were scored based on the degree of neutrophil invasion. It was observed that the degree of inflammation in the intestinal mucosa of rats dissected on day 11 was more severe than in rats dissected on day 15, which may be related to self-recovery of the intestinal flora. The dysregulation of host-microbiota interactions in the gut is a pivotal characteristic of Crohn’s disease [73]. However, whether commensals and/or the dysbiotic microbiota associated with pathology in humans are causally involved in Crohn’s pathogenesis remains controversial [73]. In the present study, the degree of inflammation of the colon and rectal tissues was investigated in antibiotic-induced SD rat enteritis models. It was found that with increasing dose or number of antibiotics used, the more severe the inflammation of the colonic and rectal tissues.(Fig 6D) This phenomenon may be associated with the release of inflammatory factors by the disordered intestinal flora [74], or with damage of intestinal mucosa caused by the release of metabolites by pathogenic microbiota, which induces humoral and cellular immunity [74, 75]. Improper use of antibiotics induces intestinal flora imbalance, which can cause changes in metabolism [46, 47] and the production of novel metabolites, such as hydrogen sulfide [76]. This altered metabolism induces changes in body fluids and cellular immunity [53], thereby destroying the intestinal mucosa of the body, leading to intestinal inflammation and the formation of intestinal ulcers, which may induce IBD [53]. The present study demonstrated gut inflammation induced by antibiotics in the proposed IBD model. In addition, the improper use of broad-spectrum antibiotics can also lead to obesity [77].

The present study has some limitations, such as a lack of information concerning the inflammatory factors with respect to intestinal mucosa necrosis, as well as the mechanisms connecting IBD and immune function within the body. In this paper, we did not explore whether enteritis caused by intestinal microbial disorder was caused by cellular immune attack or humoral immune attack. At the same time, we had not studied the signaling pathway of this mechanism. For example, we did not analyze the changes of CD4+ and CD8+ in tissues of different groups and we did not elaborate whether the mechanism of intestinal inflammation was similar to that of DSS and TNBS mice, eg. whether T helper cells played a role in information transmission. These may lead us to the direction of further research in the future. Additionally, flow cytometric analysis was not performed to examine immune cell populations in the different groups. Meanwhile, we did not compare and correlate the inflammatory markers in the histology with the altered cytokine mRNA expression.

## Conclusion

The present study indicated that it is feasible to establish an antibiotic-induced IBD model in SD rats, which may provide useful models for clinical IBD research.

## Supporting information

S1 Table Sequences of primers used for quantitative PCR. (DOCX)

S2 Table Comparison of basic status of rats across the study period (days 1,3,5,7 and 9). (DOCX)

S3 Table Comparison of total nine microbiota across days 1, 3, 5, 7, 9, 11 and 14 between the experimental groups. (DOCX)

S4 Table Comparison of inflammation factors between groups. (DOCX)

S5 Table Comparisons of inflammation scores in colon and rectum of animals. (DOCX)

S1 Raw image Fecal bacterial content and gut inflammation scores. (A) Comparison of bacterial amplification via quantitative PCR in groups A, C and F;(B) *Bacteroides*, *F*. *prausnitzii* and *D*. *invisus* agarose electrophoresis; (C)Comparison of colonic and rectal tissue inflammation scores on day 11 and day 15;(D)Comparison of colonic and rectal tissue inflammation scores of control group vs experiment groups respectively,*P<0.05, **P<0.01).
(PDF)
