# Supplementary material for: Establishment and evaluation of a specific antibiotic-induced inflammatory bowel disease model in rats
Source: PLoS One. 2022 Feb 22;17(2):e0264194. doi: 10.1371/journal.pone.0264194 (PMC8863245; doi:10.1371/journal.pone.0264194)
Supplement: S1 Table — (DOCX) [file pone.0264194.s001.docx]

S1 Table . Sequences of primers used for quantitative PCR.

| Target bacteria | Forward sequence (5’-3’) | Reverse sequence (5’-3’) | Product size, bp | Accession no. |
| --- | --- | --- | --- | --- |
| *Bacteroides* | TTAAGTATTCCACCTGGGGAGT | TTAAGCCCGGGTAAGGTTCCT | 156 | CR626927 |
| *Faecalibacterium prausnitzii* | CACGGCTCTGGAAATCTATGT | GCACAATGAGCATACCGAGTT | 140 | NZ_PXUP01000071 |
| *Dialister invisus* | AGACGGAAACGACTGCTAATACC | CAGCTAATCAGACGCAAACCC | 116 | LT223661 |
