# Supplementary material for: Establishment and evaluation of a specific antibiotic-induced inflammatory bowel disease model in rats
Source: PLoS One. 2022 Feb 22;17(2):e0264194. doi: 10.1371/journal.pone.0264194 (PMC8863245; doi:10.1371/journal.pone.0264194)
Supplement: S2 Table — (DOCX) [file pone.0264194.s002.docx]

S2 Table . Comparison of basic status of rats across the study period (days 1,3,5,7 and 9).

| Property | Group A | Group B | Group C | Group D | Group E | Group F | Group G | F-value | P-value |
| --- | --- | --- | --- | --- | --- | --- | --- | --- | --- |
| Weight, g | 204.02±13.5 | 203.31±17.2 | 208.09±22.15 | 188.13±24.05 | 209.00±18.58 | 210.70±19.18 | 212.12±20.31 | 2.313 | 0.040^a^ |
| Food intake, g | 19.02±3.63 | 19.58±2.83 | 18.93±2.75 | 15.47±5.66 | 18.80±2.11 | 20.31±3.57 | 20.66±3.13 | 2.807 | 0.016^a^ |
| Water intake, g | 38.86±4.73 | 49.76±11.31 | 44.40±9.24 | 41.96±18.95 | 60.33±9.02 | 52.68±10.23 | 53.47±7.84 | 6.213 | <0.001^b^ |
| Stool in 2 h, g | 0.96±0.48 | 2.20±1.38 | 2.23±1.09 | 2.47±1.53 | 2.82±2.01 | 3.09±1.79 | 2.33±1.55 | 3.043 | 0.009^a^ |

^a^P<0.05, ^b^P<0.001. Data are presented as the mean ± SD (n=12/group).
