# Supplementary material for: Establishment and evaluation of a specific antibiotic-induced inflammatory bowel disease model in rats
Source: PLoS One. 2022 Feb 22;17(2):e0264194. doi: 10.1371/journal.pone.0264194 (PMC8863245; doi:10.1371/journal.pone.0264194)
Supplement: S3 Table — (DOCX) [file pone.0264194.s003.docx]

S3 Table . Comparison of total nine microbiota across days 1, 3, 5, 7, 9, 11 and 14 between the experimental groups.

| Group | Total microbiota, x10^6^ (95% CI) | P-value vs. A |
| --- | --- | --- |
| A | 1376.7±3683.8 (562.1-2191.3) | NA |
| B | 1260.3±1498.7 (355.7-1018.5) | 0.546 |
| C | 1474.9±4187.6 (549.0-2400.8) | 0.872 |
| D | 478.2±1758.1 (65.0-891.3) | <0.001^a^ |
| E | 664.5±1567.9 (317.8-1011.2) | <0.001^a^ |
| F | 403.8±1172.0 (144.6-662.9) | <0.001^a^ |
| G | 568.7±567.3.(212.8-156.7) | <0.001 |

^a^P<0.001. Data are presented as the mean ± SD (n=12/group). CI, confidence interval.
