# Supplementary material for: Establishment and evaluation of a specific antibiotic-induced inflammatory bowel disease model in rats
Source: PLoS One. 2022 Feb 22;17(2):e0264194. doi: 10.1371/journal.pone.0264194 (PMC8863245; doi:10.1371/journal.pone.0264194)
Supplement: S4 Table — (DOCX) [file pone.0264194.s004.docx]

S4 Table . Comparison of inflammation factors between groups.

| Factor | Group A (95% CI) | Group B (95% CI) | Group C (95% CI) | Group D (95% CI) | Group E (95% CI) | Group F (95% CI) | Group G (95% CI) | F-value | P-value |
| --- | --- | --- | --- | --- | --- | --- | --- | --- | --- |
| TNF-α, pg/ml | 179.1±25.9 (162.7-195.8) | 207.0±12.0 (199.4-214.6) | 244.1±10.8 (237.2-251.0) | 272.3±18.3 (260.7-283.9) | 276.0±8.3 (270.8-281.3) | 311.8±9.0 (306.0-317.9) | 361.4±12.2 (353.6-369.2) | 204.89 | <0.001 |
| IL-1β, pg/ml | 65.8±19.2 (53.5-78.0) | 100.3±8.4 (94.9-105.6) | 130.6±12.5 (122.7-138.6) | 178.2±11.4 (171.0-185.4) | 218.8±36.5 (195.7-242.1) | 308.2±12.6 (300.2-216.2) | 345.8±22.3 (331.7-360.0) | 341.57 | <0.001 |
| IL-6, pg/ml | 124.0±24.8 (108.2-139.8) | 172.2±19.0 (160.1-184.3) | 222.0±27.9 (204.2-239.7) | 276.0±16.8 (265.3-286.6) | 308.7±7.3 (304.0-313.3) | 352.0±11.2 (344.8-359.1) | 414.2±15.5 (404.4-424.1) | 354.15 | <0.001 |
| CRP, pg/ml | 9.8±2.8 (8.0-11.6) | 15.9±2.2 (14.5-17.3) | 25.7±2.7 (24.0-27.4) | 35.9±1.8 (34.7-36.9) | 48.7±1.9 (47.5-49.8) | 57.2±1.6 (56.2-58.2) | 71.2±2.8 (69.4-72.9) | 1153.51 | <0.001 |

Data are presented as the mean ± standard deviation. CRP, C-reactive protein; CI, confidence interval.
