# Supplementary material for: Establishment and evaluation of a specific antibiotic-induced inflammatory bowel disease model in rats
Source: PLoS One. 2022 Feb 22;17(2):e0264194. doi: 10.1371/journal.pone.0264194 (PMC8863245; doi:10.1371/journal.pone.0264194)
Supplement: S5 Table — (DOCX) [file pone.0264194.s005.docx]

S5 Table. Comparisons of inflammation scores in colon and rectum of animals.

| A, Colon | | |
| --- | --- | --- |
| Group | Inflammation score | P-value vs. A |
| A | Reference | NA |
| B | 2.500 (2.225-2.625) | 0.002^a^ |
| C | 3.700 (3.575-3.975) | <0.001^b^ |
| D | 8.500 (8.500-8.625) | <0.001^b^ |
| E | 6.450 (5.400-6.750) | <0.001^b^ |
| F | 9.500 (9.475-9.625) | <0.001^b^ |
| G | 10.550 (10.375-11.525) | <0.001^b^ |
| B, Rectum | | |
| Group | Inflammation score | P-value vs. A |
| A | Reference | NA |
| B | 3.650 (3.475-3.825) | 0.002^a^ |
| C | 5.650 (5.575-5.825) | <0.001^b^ |
| D | 7.350 (7.275-7.825) | <0.001^b^ |
| E | 6.500 (6.375-6.625) | <0.001^b^ |
| F | 9.650 (9.475-9.825) | <0.001^b^ |
| G | 10.550 (10.475-10.775) | <0.001^b^ |

^a^P<0.05, ^b^P<0.001. Data are presented as the median (interquartile range).
