# Supplementary material for: Establishment and evaluation of a specific antibiotic-induced inflammatory bowel disease model in rats
Source: PLoS One. 2022 Feb 22;17(2):e0264194. doi: 10.1371/journal.pone.0264194 (PMC8863245; doi:10.1371/journal.pone.0264194)
Supplement: S1 Raw image — (A) Comparison of bacterial amplification via quantitative PCR in groups A, C and F;(B) Bacteroides, F. prausnitzii and D. invisus agarose electrophoresis; (C)Comparison of colonic and rectal tissue inflammation scores on day 11 and day 15;(D)Comparison of colonic and rectal tissue inflammation scores of control group vs experiment groups respectively,*P<0.05, **P<0.01). (PDF) [file pone.0264194.s006.pdf]

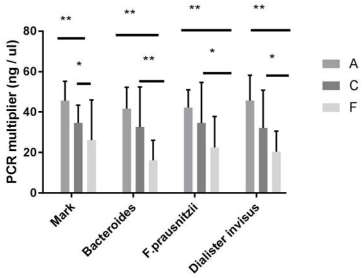
**
*
**
**
*
**
**
*
PCR multiplier (ng / ul)
80
60
40
20
0
Mark
Bacteroides
F.prausnitzii
Dialister invisus
A
C
F

A

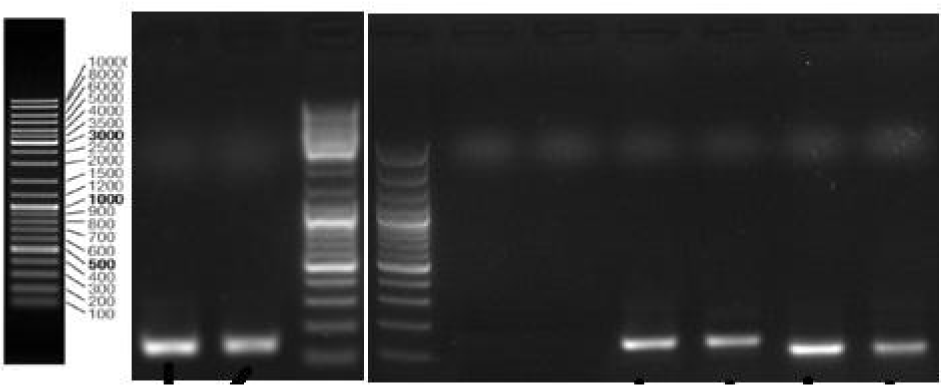
10000
8000
6000
5000
4000
3500
3000
2500
2000
1500
1200
1000
900
800
700
600
500
400
300
200
100

B

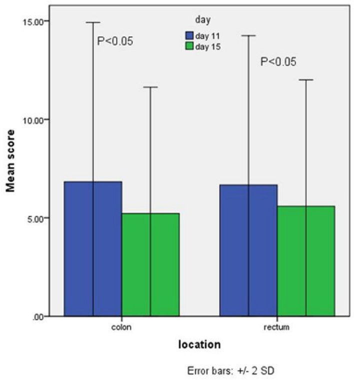
day
day 11
day 15
P<0.05
P<0.05
Mean score
15.00
10.00
5.00
.00
colon
rectum
location
Error bars: +/- 2 SD

C

D

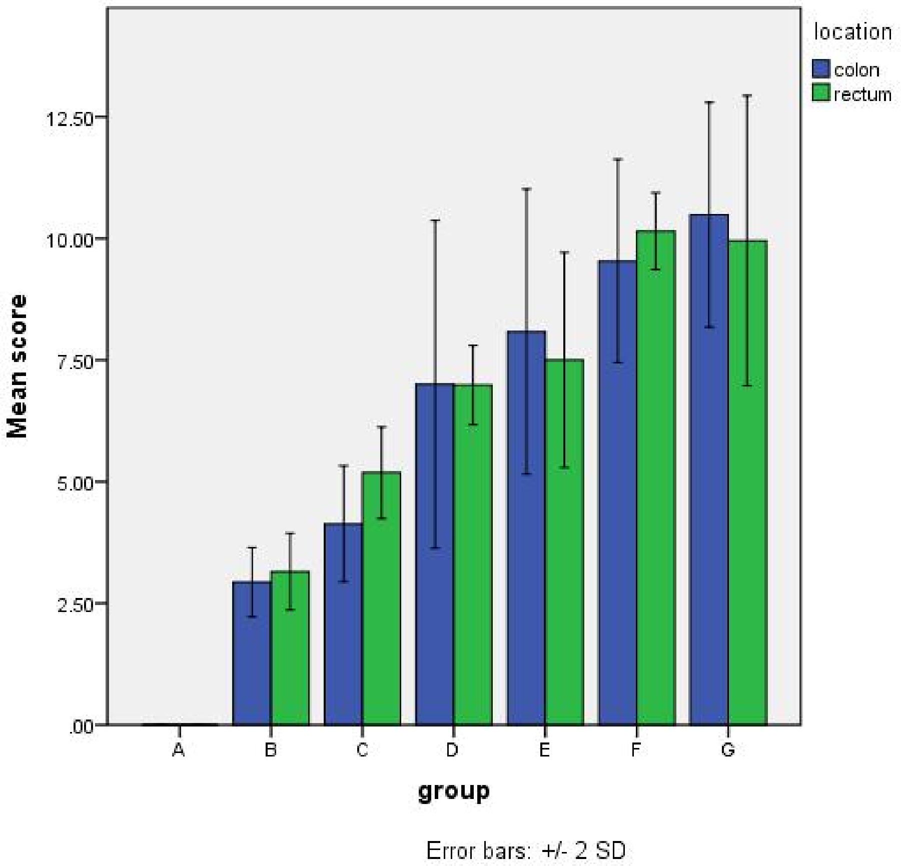

S1_raw_imagine， **Fecal bacterial content and gut inflammation scores.** (A) Comparison of bacterial amplification via quantitative PCR in groups A, C and F;(B) *Bacteroides*, *F. prausnitzii* and *D. invisus* agarose electrophoresis; (C)Comparison of colonic and rectal tissue inflammation scores on day 11 and day 15;(D)Comparison of colonic and rectal tissue inflammation scores of control group vs experiment groups respectively,*P<0.05, **P<0.01).
